# Metabolic adaptation to weight loss: implications for the athlete

**DOI:** 10.1186/1550-2783-11-7

**Published:** 2014-02-27

**Authors:** Eric T Trexler, Abbie E Smith-Ryan, Layne E Norton

**Affiliations:** 1 Department of Exercise and Sport Science, University of North Carolina at Chapel Hill, 209 Fetzer Hall, CB# 8700, Chapel Hill, NC 27599-8700, USA; 2 BioLayne LLC, Tampa, FL, USA

**Keywords:** Weight loss, Energy restriction, Body composition, Energy expenditure, Metabolic rate, Energy deficit, Weight maintenance, Uncoupling proteins, Mitochondrial efficiency

## Abstract

Optimized body composition provides a competitive advantage in a variety of sports. Weight reduction is common among athletes aiming to improve their strength-to-mass ratio, locomotive efficiency, or aesthetic appearance. Energy restriction is accompanied by changes in circulating hormones, mitochondrial efficiency, and energy expenditure that serve to minimize the energy deficit, attenuate weight loss, and promote weight regain. The current article reviews the metabolic adaptations observed with weight reduction and provides recommendations for successful weight reduction and long term reduced-weight maintenance in athletes.

## Introduction

In a variety of competitive sports, it is considered advantageous to achieve low levels of body fat while retaining lean body mass. The metabolic effects of this process have been given little context within athletics, such as physique sports (i.e. bodybuilding, figure), combat sports (i.e. judo, wrestling), aesthetic sports (i.e. gymnastics, figure skating), and endurance sports. Previous literature has documented cases of male bodybuilders reducing body fat to less than 5% of total body mass [1,2], and studies documenting physiological profiles of male wrestlers [3] and judo athletes [4] present body fat ranges that extend below 5%. A study on elite female gymnasts and runners reported an average body fat percentage (BF%) of 13.72% for the entire sample, with subgroups of middle-distance runners and artistic gymnasts averaging 12.18% and 12.36%, respectively [5]. Elite female runners have also reported percent body fat levels below 10% [6]. Energy deficits and extremely low levels of body fat present the body with a significant physiological challenge. It has been well documented that weight loss and energy restriction result in a number of homeostatic metabolic adaptations aimed at decreasing energy expenditure, improving metabolic efficiency, and increasing cues for energy intake [7-9]. While the unfavorable endocrine effects of contest preparation have been documented in male bodybuilders [1,2,10], anecdotal reports from physique athletes also describe a state in which metabolic rate has slowed to an extent that exceeds the predicted magnitude, making weight loss increasingly difficult despite low caloric intakes and high training volumes. Although such reports could potentially be related to inaccurate dietary reporting [11,12], these claims may be substantiated by a number of metabolic adaptations to weight loss, including adaptive thermogenesis [13-15], increased mitochondrial efficiency [16-19], and hormonal alterations that favor decreased energy expenditure, decreased satiety, and increased hunger [1,2,10]. As a dieting phase progresses, such adaptations may threaten dietary adherence, make further weight loss increasingly difficult, and predispose the individual to rapid weight regain following the cessation of the diet. Although data documenting the attainment and recovery from extreme changes in body composition is limited, the present article aims to investigate the condition of metabolic adaptation described by competitors and identify potential mechanisms to explain such a phenomenon.

### The endocrine response to an energy deficit

A number of hormones play prominent roles in the regulation of body composition, energy intake, and energy expenditure. The hormones of the thyroid gland, particularly triiodothyronine (T3), are known to play an important and direct role in regulating metabolic rate. Increases in circulating thyroid hormones are associated with an increase in the metabolic rate, whereas lowered thyroid levels result in decreased thermogenesis and overall metabolic rate [20]. Leptin, synthesized primarily in adipocytes, functions as an indicator of both short and long-term energy availability; short-term energy restriction and lower body fat levels are associated with decreases in circulating leptin. Additionally, higher concentrations of leptin are associated with increased satiety and energy expenditure [21]. Insulin, which plays a crucial role in inhibiting muscle protein breakdown [22] and regulating macronutrient metabolism, is considered another “adiposity signal” [23]. Similar to leptin, high levels of insulin convey a message of energy availability and are associated with an anorexigenic effect. Conversely, the orexigenic hormone ghrelin functions to stimulate appetite and food intake, and has been shown to increase with fasting, and decrease after feeding [24]. Testosterone, known primarily for its role in increasing muscle protein synthesis and muscle mass [22], may also play a role in regulating adiposity [25]. Changes in fat mass have been inversely correlated with testosterone levels, and it has been suggested that testosterone may repress adipogenesis [25]. More research is needed to delineate the exact mechanism (s) by which testosterone affects adiposity. Cortisol, a glucocorticoid that influences macronutrient metabolism, has been shown to induce muscle protein breakdown [22], and increased plasma cortisol within the physiologic range has increased proteolysis in healthy subjects [26]. Evidence also suggests that glucocorticoids may inhibit the action of leptin [27].

Results from a number of studies indicate a general endocrine response to hypocaloric diets that promotes increased hunger, reduces metabolic rate, and threatens the maintenance of lean mass. Studies involving energy restriction, or very low adiposity, report decreases in leptin [1,10,28], insulin [1,2], testosterone [1,2,28], and thyroid hormones [1,29]. Subsequently, increases in ghrelin [1,10] and cortisol [1,30,31] have been reported with energy restriction. Further, there is evidence to suggest that unfavorable changes in circulating hormone levels persist as subjects attempt to maintain a reduced body weight, even after the cessation of active weight loss [32,33].

Low energy intake and minimal body fat are perceived as indicators of energy unavailability, resulting in a homeostatic endocrine response aimed at conserving energy and promoting energy intake. It should be noted that despite alterations in plasma levels of anabolic and catabolic hormones, losses of lean body mass (LBM) often fail to reach statistical significance in studies on bodybuilding preparation [1,2]. Although the lack of significance may relate to insufficient statistical power, these findings may indicate that unfavorable, hormone-mediated changes in LBM can potentially be attenuated by sound training and nutritional practices. Previous research has indicated that structured resistance training [34] and sufficient protein intake [35-37], both commonly employed in bodybuilding contest preparation, preserve LBM during energy restriction. Further, Maestu et al. speculate that losses in LBM are dependent on the magnitude of weight loss and degree of adiposity, as the subjects who lost the greatest amount of weight and achieved the lowest final body fat percentage in the study saw the greatest losses of LBM [2]. The hormonal environment created by low adiposity and energy restriction appears to promote weight regain and threaten lean mass retention, but more research is needed to determine the chronic impact of these observed alterations in circulating anabolic and catabolic hormones.

### Weight loss and metabolic rate

**Figure 1:**
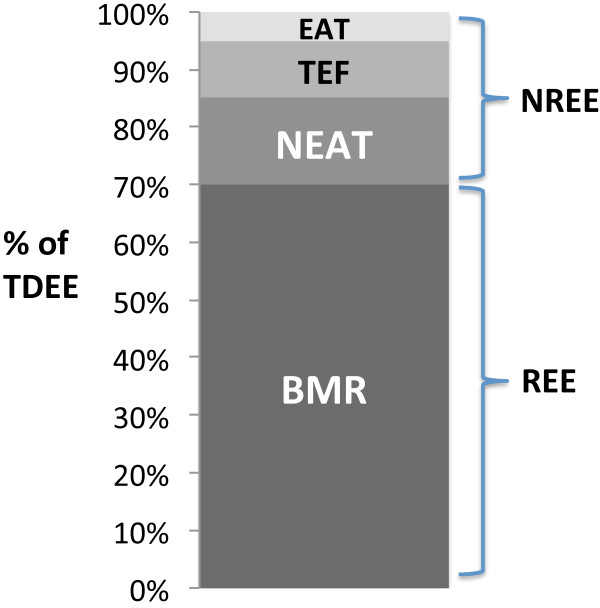
**Components of total daily energy expenditure (TDEE).** BMR = basal metabolic rate; NEAT = non-exercise activity thermogenesis; TEF = thermic effect of food; EAT = exercise activity thermogenesis; REE = resting energy expenditure; NREE = non-resting energy expenditure. Adapted from Maclean et al., 2011.

An individual’s total daily energy expenditure (TDEE) is comprised of a number of distinct components (Figure 1). The largest component, resting energy expenditure (REE), refers to the basal metabolic rate (BMR) [8]. The other component, known as non-resting energy expenditure (NREE), can be further divided into exercise activity thermogenesis (EAT), non-exercise activity thermogenesis (NEAT), and the thermic effect of food (TEF) [8].

Metabolic rate is dynamic in nature, and previous literature has shown that energy restriction and weight loss affect numerous components of energy expenditure. In weight loss, TDEE has been consistently shown to decrease [38,39]. Weight loss results in a loss of metabolically active tissue, and therefore decreases BMR [38,39]. Interestingly, the decline in TDEE often exceeds the magnitude predicted by the loss of body mass. Previous literature refers to this excessive drop in TDEE as adaptive thermogenesis, and suggests that it functions to promote the restoration of baseline body weight [13-15]. Adaptive thermogenesis may help to partially explain the increasing difficulty experienced when weight loss plateaus despite low caloric intake, and the common propensity to regain weight after weight loss.

Exercise activity thermogenesis also drops in response to weight loss [40-42]. In activity that involves locomotion, it is clear that reduced body mass will reduce the energy needed to complete a given amount of activity. Interestingly, when external weight is added to match the subject’s baseline weight, energy expenditure to complete a given workload remains below baseline [41]. It has been speculated that this increase in skeletal muscle efficiency may be related to the persistent hypothyroidism and hypoleptinemia that accompany weight loss, resulting in a lower respiratory quotient and greater reliance on lipid metabolism [43].

The TEF encompasses the energy expended in the process of ingesting, absorbing, metabolizing, and storing nutrients from food [8]. Roughly 10% of TDEE is attributed to TEF [44,45], with values varying based on the macronutrient composition of the diet. While the relative magnitude of TEF does not appear to change with energy restriction [46], such dietary restriction involves the consumption of fewer total calories, and therefore decreases the absolute magnitude of TEF [41,46]. NEAT, or energy expended during “non-exercise” movement such as fidgeting or normal daily activities, also decreases with an energy deficit [47]. There is evidence to suggest that spontaneous physical activity, a component of NEAT, is decreased in energy restricted subjects, and may remain suppressed for some time after subjects return to *ad libitum* feeding [29]. Persistent suppression of NEAT may contribute to weight regain in the post-diet period.

In order to manipulate an individual’s body mass, energy intake must be adjusted based on the individual’s energy expenditure. In the context of weight loss or maintaining a reduced body weight, this process is complicated by the dynamic nature of energy expenditure. In response to weight loss, reductions in TDEE, BMR, EAT, NEAT, and TEF are observed. Due to adaptive thermogenesis, TDEE is lowered to an extent that exceeds the magnitude predicted by losses in body mass. Further, research indicates that adaptive thermogenesis and decreased energy expenditure persist after the active weight loss period, even in subjects who have maintained a reduced body weight for over a year [14,48]. These changes serve to minimize the energy deficit, attenuate further loss of body mass, and promote weight regain in weight-reduced subjects.

### Adaptations in mitochondrial efficiency

A series of chemical reactions must take place to derive ATP from stored and ingested energy substrates. In aerobic metabolism, this process involves the movement of protons across the inner mitochondrial membrane. When protons are transported by ATP synthase, ATP is produced. Protons may also leak across the inner membrane by way of uncoupling proteins (UCPs) [49]. In this “uncoupled respiration”, energy substrate oxidation and oxygen consumption occur, but the process does not yield ATP. Proton leak is a significant contributor to energy expenditure, accounting for roughly 20-30% of BMR in rats [50-52].

In the condition of calorie restriction, proton leak is reduced [16-19]. Uncoupling protein-1 and UCP-3, the primary UCPs of brown adipose tissue (BAT) and skeletal muscle [53], are of particular interest due to their potentially significant roles in energy expenditure and uncoupled thermogenesis. Skeletal muscle’s large contribution to energy expenditure [54] has directed attention toward literature reporting decreases in UCP-3 expression in response to energy restriction [55,56]. Decreased UCP-3 expression could potentially play a role in decreasing energy expenditure, and UCP-3 expression has been negatively correlated with body mass index and positively correlated with metabolic rate during sleep [57]. Despite these correlations, more research is needed to determine the function and physiological relevance of UCP-3 [58], as contradictory findings regarding UCP-3 and weight loss have been reported [18].

Uncoupling Protein-1 appears to play a pivotal role in the uncoupled thermogenic activity of BAT [59]. Energy restriction has been shown to decrease BAT activation [60] and UCP-1 expression [61], indicating an increase in metabolic efficiency. Along with UCP-1 expression, thyroid hormone and leptin affect the magnitude of uncoupled respiration in BAT. Thyroid hormone (TH) and leptin are associated with increased BAT activation, whereas glucocorticoids oppose the BAT-activating function of leptin [59]. Evidence indicates that TH plays a prominent role in modulating the magnitude of proton leak [53], with low TH levels associated with decreased proton leak [62]. The endocrine response to energy restriction, including increased cortisol and decreased TH and leptin [1,10,28-31], could potentially play a regulatory role in uncoupled respiration in BAT. It is not clear if decreases in proton leak and UCP expression persist until weight reverts to baseline, but there is evidence to suggest a persistent adaptation [19,55,56], which mirrors the persistent downregulation of TH and leptin [32,33].

Changes observed in proton leak, UCP expression, and circulating hormones appear to influence metabolic efficiency and energy expenditure. In the context of energy restriction, the observed changes are likely to make weight loss increasingly challenging and promote weight regain. It has been reported that females have more BAT than males [63], and that energy-restricted female rats see greater decreases in BAT mass and UCP-1 than males [64], indicating a potential sex-related difference in uncoupled respiration during weight loss. Subjects identified as “diet-resistant” show decreased proton leak and UCP-3 expression compared to “diet-responsive” subjects during maintenance of a reduced bodyweight [65]. More research is needed to determine if these differential responses to hypocaloric diets make sustained weight loss more difficult for females and certain predisposed “diet-resistant” individuals. While future research may improve our understanding of the magnitude and relative importance of mitochondrial adaptations to energy restriction, current evidence suggests that increased mitochondrial efficiency, and a decline in uncoupled respiration, might serve to decrease the energy deficit in hypocaloric conditions, making weight maintenance and further weight reduction more challenging.

### Practical applications for weight loss in athletes

Hypocaloric diets induce a number of adaptations that serve to prevent further weight loss and conserve energy. It is likely that the magnitude of these adaptations are proportional to the size of the energy deficit, so it is recommended to utilize the smallest possible deficit that yields appreciable weight loss. This may decrease the rate of weight loss, but attenuate unfavorable adaptations that challenge successful reduction of fat mass. Weight reduction should be viewed as a stepwise process in this context; as weight loss begins to plateau, energy intake or expenditure should be adjusted to “re-open” the energy deficit. Large caloric deficits are also likely to induce greater losses of LBM [66,67] and compromise athletic performance and recovery [68,69], which are of critical importance to athletes. Participation in a structured resistance training program [34] and sufficient protein intake [35-37] are also likely to attenuate losses in LBM. Additionally, high protein diets (≥25%PRO) are associated with increased satiety and thermogenesis, making them a better option for the calorie-restricted athlete [70].

**Figure 2:**
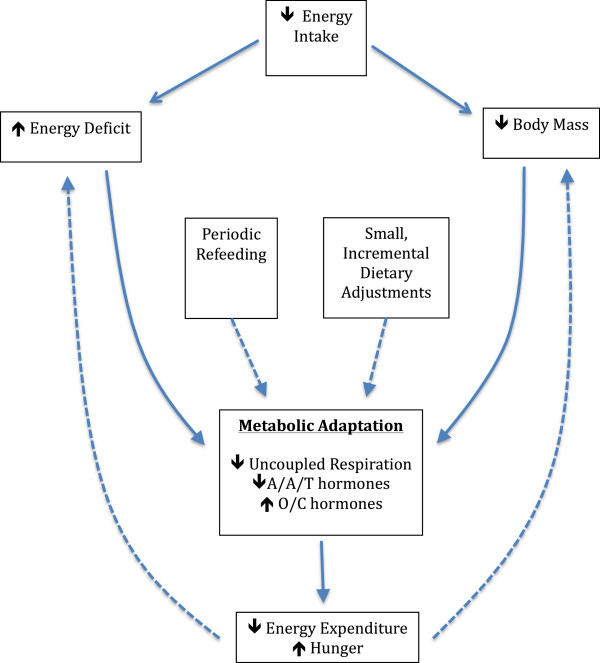
**A theoretical model of metabolic adaptation and potential strategies to attenuate adaptations.** A/A/T hormones = Anabolic, Anorexigenic, and Thermogenic hormones; O/C hormones = Orexigenic and Catabolic hormones. Dotted lines represent inhibition.

In the world of physique sports, periodic “refeeding” has become common in periods of extended dieting. A refeed consists of a brief overfeeding period in which caloric intake is raised slightly above maintenance levels, and the increase in caloric intake is predominantly achieved by increasing carbohydrate consumption. While studies have utilized refeeding protocols that last three days [71,72], physique athletes such as bodybuilders and figure competitors often incorporate 24-hour refeeds, once or twice per week. The proposed goal of periodic refeeding is to temporarily increase circulating leptin and stimulate the metabolic rate. There is evidence indicating that leptin is acutely responsive to short-term overfeeding [72], is highly correlated with carbohydrate intake [71,73], and that pharmacological administration of leptin reverses many unfavorable adaptations to energy restriction [33]. While interventions have shown acute increases in leptin from short-term carbohydrate overfeeding, the reported effect on metabolic rate has been modest [71]. Dirlewanger et al. reported a 7% increase in TDEE; this increase amounts to approximately 138 kilocalories of additional energy expenditure, of which 36 kilocalories can be attributed to the thermic effect of carbohydrate intake [71]. More research is needed to determine if acute bouts of refeeding are an efficacious strategy for improving weight loss success during prolonged hypocaloric states. A theoretical model of metabolic adaptation and potential strategies to attenuate adaptations is presented in Figure 2.

In the period shortly after cessation of a restrictive diet, body mass often reverts toward pre-diet values [29,74,75]. This body mass is preferentially gained as fat mass, in a phenomenon known as post-starvation obesity [29]. While many of the metabolic adaptations to weight loss persist, a dramatic increase in energy intake results in rapid accumulation of fat mass. It is common for individuals to “overshoot” their baseline level of body fat, and leaner individuals (including many athletes) may be more susceptible to overshooting than obese individuals [74,75]. In such a situation, the individual may increase body fat beyond baseline levels, yet retain a metabolic rate that has yet to fully recover. There is evidence to suggest that adipocyte hyperplasia may occur early in the weight-regain process [76], and that repeated cycles of weight loss and regain by athletes in sports with weight classes are associated with long-term weight gain [77]. Therefore, athletes who aggressively diet for a competitive season and rapidly regain weight may find it more challenging to achieve optimal body composition in subsequent seasons.

To avoid rapid fat gain following the cessation of a diet, “reverse dieting” has also become popular among physique athletes. Such a process involves slowly increasing caloric intake in a stepwise fashion. In theory, providing a small caloric surplus might help to restore circulating hormone levels and energy expenditure toward pre-diet values, while closely matching energy intake to the recovering metabolic rate in an effort to reduce fat accretion. Ideally, such a process would eventually restore circulating hormones and metabolic rate to baseline levels while avoiding rapid fat gain. While anecdotal reports of successful reverse dieting have led to an increase in its popularity, research is needed to evaluate its efficacy.

### Limitations

Although there is a substantial body of research on metabolic adaptations to weight loss, the majority of the research has utilized animal models or subjects that are sedentary and overweight/obese. Accordingly, the current article is limited by the need to apply this data to an athletic population. If the adaptations described in obese populations serve to conserve energy and attenuate weight loss as a survival mechanism, one might speculate that the adaptations may be further augmented in a leaner, more highly active population. Another limitation is the lack of research on the efficacy of periodic refeeding or reverse dieting in prolonged weight reduction, or in the maintenance of a reduced bodyweight. Until such research is available, these anecdotal methods can only be evaluated from a mechanistic and theoretical viewpoint.

## Conclusion

Weight loss is a common practice in a number of sports. Whether the goal is a higher strength-to-mass ratio, improved aesthetic presentation, or more efficient locomotion, optimizing body composition is advantageous to a wide variety of athletes. As these athletes create an energy deficit and achieve lower body fat levels, their weight loss efforts will be counteracted by a number of metabolic adaptations that may persist throughout weight maintenance. Changes in energy expenditure, mitochondrial efficiency, and circulating hormone concentrations work in concert to attenuate further weight loss and promote the restoration of baseline body mass. Athletes must aim to minimize the magnitude of these adaptations, preserve LBM, and adequately fuel performance and recovery during weight reduction. To accomplish these goals, it is recommended to approach weight loss in a stepwise, incremental fashion, utilizing small energy deficits to ensure a slow rate of weight loss. Participation in a structured resistance training program and adequate protein intake are also imperative. More research is needed to verify the efficacy of periodic refeeding and reverse dieting in supporting prolonged weight reduction and attenuating post-diet fat accretion.

## Abbreviations

BAT: Brown adipose tissue; BF%: Body fat percentage; BMR: Basal metabolic rate; EAT: Exercise activity thermogenesis; LBM: Lean body mass; NEAT: Non-exercise activity thermogenesis; NREE: Non-resting energy expenditure; REE: Resting energy expenditure; TDEE: Total daily energy expenditure; TEF: Thermic effect of food; TH: Thyroid Hormone; T3: Triiodothyronine; UCP: Uncoupling protein.

## Competing interests

The authors declare that they have no competing interests.

## Authors’ contributions

ETT conceived of the review topic and drafted the manuscript. AES conceived, drafted and revised the manuscript. LEN helped to draft and revise the manuscript. All authors read and approved the final manuscript.

## References

[B1] Rossow LM Fukuda DH Fahs CA Loenneke JP Stout JR Natural bodybuilding competition preparation and recovery: a 12-month case study Int J Sports Physiol Perform 2013 8 582 592 23412685 10.1123/ijspp.8.5.582

[B2] Maestu J Eliakim A Jurimae J Valter I Jurimae T Anabolic and catabolic hormones and energy balance of the male bodybuilders during the preparation for the competition J Strength Cond Res 2010 24 1074 1081 10.1519/JSC.0b013e3181cb6fd3 20300017

[B3] Yoon J Physiological profiles of elite senior wrestlers Sports Med 2002 32 225 233 10.2165/00007256-200232040-00002 11929352

[B4] Franchini E Del Vecchio FB Matsushigue KA Artioli GG Physiological profiles of elite judo athletes Sports Med 2011 41 147 166 10.2165/11538580-000000000-00000 21244106

[B5] Deutz RC Benardot D Martin DE Cody MM Relationship between energy deficits and body composition in elite female gymnasts and runners Med Sci Sports Exerc 2000 32 659 668 10731010 10.1097/00005768-200003000-00017

[B6] Wilmore JH Brown CH Davis JA Body physique and composition of the female distance runner Ann N Y Acad Sci 1977 301 764 776 10.1111/j.1749-6632.1977.tb38245.x 270950

[B7] Dulloo AG Jacquet J Adaptive reduction in basal metabolic rate in response to food deprivation in humans: a role for feedback signals from fat stores Am J Clin Nutr 1998 68 599 606 9734736 10.1093/ajcn/68.3.599

[B8] Maclean PS Bergouignan A Cornier MA Jackman MR Biology’s response to dieting: the impetus for weight regain Am J Physiol Regul Integr Comp Physiol 2011 301 R581 R600 10.1152/ajpregu.00755.2010 21677272 PMC3174765

[B9] MacLean PS Higgins JA Jackman MR Johnson GC Fleming-Elder BK Wyatt HR Melanson EL Hill JO Peripheral metabolic responses to prolonged weight reduction that promote rapid, efficient regain in obesity-prone rats Am J Physiol Regul Integr Comp Physiol 2006 290 R1577 R1588 10.1152/ajpregu.00810.2005 16455763

[B10] Maestu J Jurimae J Valter I Jurimae T Increases in ghrelin and decreases in leptin without altering adiponectin during extreme weight loss in male competitive bodybuilders Metabolism 2008 57 221 225 10.1016/j.metabol.2007.09.004 18191052

[B11] Lichtman SW Pisarska K Berman ER Pestone M Dowling H Offenbacher E Weisel H Heshka S Matthews DE Heymsfield SB Discrepancy between self-reported and actual caloric intake and exercise in obese subjects N Engl J Med 1992 327 1893 1898 10.1056/NEJM199212313272701 1454084

[B12] Garriguet D Under-reporting of energy intake in the Canadian community health survey Health Rep 2008 19 37 45 19226926

[B13] Doucet E St-Pierre S Almeras N Despres JP Bouchard C Tremblay A Evidence for the existence of adaptive thermogenesis during weight loss Br J Nutr 2001 85 715 723 10.1079/BJN2001348 11430776

[B14] Rosenbaum M Hirsch J Gallagher DA Leibel RL Long-term persistence of adaptive thermogenesis in subjects who have maintained a reduced body weight Am J Clin Nutr 2008 88 906 912 18842775 10.1093/ajcn/88.4.906

[B15] Rosenbaum M Leibel RL Adaptive thermogenesis in humans Int J Obes 2010 34 Suppl 1 S47 S55 10.1038/ijo.2010.184 PMC3673773 20935667

[B16] Asami DK McDonald RB Hagopian K Horwitz BA Warman D Hsiao A Warden C Ramsey JJ Effect of aging, caloric restriction, and uncoupling protein 3 (UCP3) on mitochondrial proton leak in mice Exp Gerontol 2008 43 1069 1076 10.1016/j.exger.2008.09.010 18852040 PMC2614627

[B17] Bevilacqua L Ramsey JJ Hagopian K Weindruch R Harper ME Effects of short- and medium-term calorie restriction on muscle mitochondrial proton leak and reactive oxygen species production Am J Physiol Regul Integr Comp Physiol 2004 286 E852 E861 10.1152/ajpendo.00367.2003 14736705

[B18] Bevilacqua L Ramsey JJ Hagopian K Weindruch R Harper ME Long-term caloric restriction increases UCP3 content but decreases proton leak and reactive oxygen species production in rat skeletal muscle mitochondria Am J Physiol Endocrinol Metab 2005 289 E429 E438 10.1152/ajpendo.00435.2004 15886224

[B19] Hagopian K Harper ME Ram JJ Humble SJ Weindruch R Ramsey JJ Long-term calorie restriction reduces proton leak and hydrogen peroxide production in liver mitochondria Am J Physiol Endocrinol Metab 2005 288 E674 E684 15562252 10.1152/ajpendo.00382.2004

[B20] Kim B Thyroid hormone as a determinant of energy expenditure and the basal metabolic rate Thyroid 2008 18 141 144 10.1089/thy.2007.0266 18279014

[B21] Margetic S Gazzola C Pegg GG Hill RA Leptin: a review of its peripheral actions and interactions Int J Obes Relat Metab Disord 2002 26 1407 1433 10.1038/sj.ijo.0802142 12439643

[B22] Rooyackers OE Nair KS Hormonal regulation of human muscle protein metabolism Annu Rev Nutr 1997 17 457 485 10.1146/annurev.nutr.17.1.457 9240936

[B23] Strohacker K McCaffery JM Maclean PS Wing RR Adaptations of leptin, ghrelin or insulin during weight loss as predictors of weight regain: a review of current literature Int J Obes 2013 1 9 http://www.nature.com/ijo/journal/vaop/ncurrent/full/ijo2013118a.html 10.1038/ijo.2013.118 PMC5357888 23801147

[B24] Ariyasu H Takaya K Tagami T Ogawa Y Hosoda K Akamizu T Suda M Koh T Natsui K Toyooka S Ariyasu H Takaya K Tagami T Ogawa Y Hosoda K Akamizu T Suda M Koh T Natsui K Toyooka S Shirakami G Usui T Shimatsu A Doi K Hosoda H Kojima M Kangawa K Nakao K Stomach is a major source of circulating ghrelin, and feeding state determines plasma ghrelin-like immunoreactivity levels in humans J Clin Endocrinol Metab 2001 86 4753 4758 10.1210/jcem.86.10.7885 11600536

[B25] De Maddalena C Vodo S Petroni A Aloisi AM Impact of testosterone on body fat composition J Cell Physiol 2012 227 3744 3748 10.1002/jcp.24096 22495883

[B26] Simmons PS Miles JM Gerich JE Haymond MW Increased proteolysis. An effect of increases in plasma cortisol within the physiologic range J Clin Invest 1984 73 412 420 10.1172/JCI111227 6365973 PMC425032

[B27] Zakrzewska KE Cusin I Sainsbury A Rohner-Jeanrenaud F Jeanrenaud B Glucocorticoids as counterregulatory hormones of leptin: toward an understanding of leptin resistance Diabetes 1997 46 717 719 10.2337/diab.46.4.717 9075817

[B28] Hagmar M Berglund B Brismar K Hirschberg AL Body composition and endocrine profile of male Olympic athletes striving for leanness Clin J Sport Med 2013 23 197 201 10.1097/JSM.0b013e31827a8809 23275346

[B29] Weyer C Walford RL Harper IT Milner M MacCallum T Tataranni PA Ravussin E Energy metabolism after 2 y of energy restriction: the biosphere 2 experiment Am J Clin Nutr 2000 72 946 953 11010936 10.1093/ajcn/72.4.946

[B30] Witbracht MG Laugero KD Van Loan MD Adams SH Keim NL Performance on the Iowa gambling task is related to magnitude of weight loss and salivary cortisol in a diet-induced weight loss intervention in overweight women Physiol Behav 2012 106 291 297 10.1016/j.physbeh.2011.04.035 21565212

[B31] Tomiyama AJ Mann T Vinas D Hunger JM Dejager J Taylor SE Low calorie dieting increases cortisol Psychosom Med 2010 72 357 364 10.1097/PSY.0b013e3181d9523c 20368473 PMC2895000

[B32] Sumithran P Prendergast LA Delbridge E Purcell K Shulkes A Kriketos A Proietto J Long-term persistence of hormonal adaptations to weight loss N Engl J Med 2011 365 1597 1604 10.1056/NEJMoa1105816 22029981

[B33] Rosenbaum M Goldsmith R Bloomfield D Magnano A Weimer L Heymsfield S Gallagher D Mayer L Murphy E Leibel RL Low-dose leptin reverses skeletal muscle, autonomic, and neuroendocrine adaptations to maintenance of reduced weight J Clin Invest 2005 115 3579 3586 10.1172/JCI25977 16322796 PMC1297250

[B34] Bryner RW Ullrich IH Sauers J Donley D Hornsby G Kolar M Yeater R Effects of resistance vs. aerobic training combined with an 800 calorie liquid diet on lean body mass and resting metabolic rate J Am Coll Nutr 1999 18 115 121 10.1080/07315724.1999.10718838 10204826

[B35] Mettler S Mitchell N Tipton KD Increased protein intake reduces lean body mass loss during weight loss in athletes Med Sci Sports Exerc 2010 42 326 337 19927027 10.1249/MSS.0b013e3181b2ef8e

[B36] Layman DK Boileau RA Erickson DJ Painter JE Shiue H Sather C Christou DD A reduced ratio of dietary carbohydrate to protein improves body composition and blood lipid profiles during weight loss in adult women J Nutr 2003 133 411 417 12566476 10.1093/jn/133.2.411

[B37] Bopp MJ Houston DK Lenchik L Easter L Kritchevsky SB Nicklas BJ Lean mass loss is associated with low protein intake during dietary-induced weight loss in postmenopausal women J Am Diet Assoc 2008 108 1216 1220 10.1016/j.jada.2008.04.017 18589032 PMC3665330

[B38] Ravussin E Burnand B Schutz Y Jequier E Energy expenditure before and during energy restriction in obese patients Am J Clin Nutr 1985 41 753 759 3984927 10.1093/ajcn/41.4.753

[B39] Leibel RL Rosenbaum M Hirsch J Changes in energy expenditure resulting from altered body weight N Engl J Med 1995 332 621 628 10.1056/NEJM199503093321001 7632212

[B40] Weigle DS Contribution of decreased body mass to diminished thermic effect of exercise in reduced-obese men Int J Obes 1988 12 567 578 3235273

[B41] Weigle DS Brunzell JD Assessment of energy expenditure in ambulatory reduced-obese subjects by the techniques of weight stabilization and exogenous weight replacement Int J Obes 1990 14 Suppl 1 69 77 discussion 77–81 2228420

[B42] Doucet E Imbeault P St-Pierre S Almeras N Mauriege P Despres JP Bouchard C Tremblay A Greater than predicted decrease in energy expenditure during exercise after body weight loss in obese men Clin Sci 2003 105 89 95 10.1042/CS20020252 12617720

[B43] Rosenbaum M Vandenborne K Goldsmith R Simoneau JA Heymsfield S Joanisse DR Hirsch J Murphy E Matthews D Segal KR Leibel RL Effects of experimental weight perturbation on skeletal muscle work efficiency in human subjects Am J Physiol Regul Integr Comp Physiol 2003 285 R183 192 12609816 10.1152/ajpregu.00474.2002

[B44] Tappy L Thermic effect of food and sympathetic nervous system activity in humans Reprod Nutr Dev 1996 36 391 397 10.1051/rnd:19960405 8878356

[B45] Ravussin E Lillioja S Anderson TE Christin L Bogardus C Determinants of 24-hour energy expenditure in man. Methods and results using a respiratory chamber J Clin Invest 1986 78 1568 1578 10.1172/JCI112749 3782471 PMC423919

[B46] Miles CW Wong NP Rumpler WV Conway J Effect of circadian variation in energy expenditure, within-subject variation and weight reduction on thermic effect of food Eur J Clin Nutr 1993 47 274 284 8491165

[B47] Levine JA Non-exercise activity thermogenesis (NEAT) Best Pract Res Clin Endocrinol Metab 2002 16 679 702 10.1053/beem.2002.0227 12468415

[B48] Leibel RL Hirsch J Diminished energy requirements in reduced-obese patients Metabolism 1984 33 164 170 10.1016/0026-0495(84)90130-6 6694559

[B49] Jastroch M Divakaruni AS Mookerjee S Treberg JR Brand MD Mitochondrial proton and electron leaks Essays Biochem 2010 47 53 67 10.1042/bse0470053 20533900 PMC3122475

[B50] Rolfe DF Brand MD Contribution of mitochondrial proton leak to skeletal muscle respiration and to standard metabolic rate Am J Physiol 1996 271 C1380 1389 8897845 10.1152/ajpcell.1996.271.4.C1380

[B51] Rolfe DF Brown GC Cellular energy utilization and molecular origin of standard metabolic rate in mammals Physiol Rev 1997 77 731 758 9234964 10.1152/physrev.1997.77.3.731

[B52] Rolfe DF Newman JM Buckingham JA Clark MG Brand MD Contribution of mitochondrial proton leak to respiration rate in working skeletal muscle and liver and to SMR Am J Physiol 1999 276 C692 699 10069997 10.1152/ajpcell.1999.276.3.C692

[B53] Thrush AB Dent R McPherson R Harper ME Implications of mitochondrial uncoupling in skeletal muscle in the development and treatment of obesity FEBS J 2013 280 5015 5029 10.1111/febs.12399 23786211

[B54] Zurlo F Larson K Bogardus C Ravussin E Skeletal muscle metabolism is a major determinant of resting energy expenditure J Clin Invest 1990 86 1423 1427 10.1172/JCI114857 2243122 PMC296885

[B55] Esterbauer H Oberkofler H Dallinger G Breban D Hell E Krempler F Patsch W Uncoupling protein-3 gene expression: reduced skeletal muscle mRNA in obese humans during pronounced weight loss Diabetologia 1999 42 302 309 10.1007/s001250051155 10096782

[B56] Vidal-Puig A Rosenbaum M Considine RC Leibel RL Dohm GL Lowell BB Effects of obesity and stable weight reduction on UCP2 and UCP3 gene expression in humans Obes Res 1999 7 133 140 10.1002/j.1550-8528.1999.tb00694.x 10102249

[B57] Schrauwen P Xia J Bogardus C Pratley RE Ravussin E Skeletal muscle uncoupling protein 3 expression is a determinant of energy expenditure in Pima Indians Diabetes 1999 48 146 149 10.2337/diabetes.48.1.146 9892236

[B58] Harper ME Dent RM Bezaire V Antoniou A Gauthier A Monemdjou S McPherson R UCP3 and its putative function: consistencies and controversies Biochem Soc Trans 2001 29 768 773 10.1042/BST0290768 11709072

[B59] Cannon B Nedergaard J Brown adipose tissue: function and physiological significance Physiol Rev 2004 84 277 359 10.1152/physrev.00015.2003 14715917

[B60] Rothwell NJ Stock MJ Effect of chronic food restriction on energy balance, thermogenic capacity, and brown-adipose-tissue activity in the rat Biosci Rep 1982 2 543 549 10.1007/BF01314214 7139069

[B61] Young JB Saville E Rothwell NJ Stock MJ Landsberg L Effect of diet and cold exposure on norepinephrine turnover in brown adipose tissue of the rat J Clin Invest 1982 69 1061 1071 10.1172/JCI110541 7068845 PMC370170

[B62] Harper ME Brand MD The quantitative contributions of mitochondrial proton leak and ATP turnover reactions to the changed respiration rates of hepatocytes from rats of different thyroid status J Biol Chem 1993 268 14850 14860 8392060

[B63] Cypess AM Lehman S Williams G Tal I Rodman D Goldfine AB Kuo FC Palmer EL Tseng YH Doria A Cypess AM Lehman S Williams G Tal I Rodman D Goldfine AB Kuo FC Palmer EL Tseng YH Doria A Kolodny GM Kahn CR Identification and importance of brown adipose tissue in adult humans N Engl J Med 2009 360 1509 1517 10.1056/NEJMoa0810780 19357406 PMC2859951

[B64] Valle A Catala-Niell A Colom B Garcia-Palmer FJ Oliver J Roca P Sex-related differences in energy balance in response to caloric restriction Am J Physiol Endocrinol Metab 2005 289 E15 22 10.1152/ajpendo.00553.2004 15701677

[B65] Harper ME Dent R Monemdjou S Bezaire V Van Wyck L Wells G Kavaslar GN Gauthier A Tesson F McPherson R Decreased mitochondrial proton leak and reduced expression of uncoupling protein 3 in skeletal muscle of obese diet-resistant women Diabetes 2002 51 2459 2466 10.2337/diabetes.51.8.2459 12145158

[B66] Chaston TB Dixon JB O’Brien PE Changes in fat-free mass during significant weight loss: a systematic review Int J Obes 2007 31 743 750 10.1038/sj.ijo.0803483 17075583

[B67] Garthe I Raastad T Refsnes PE Koivisto A Sundgot-Borgen J Effect of two different weight-loss rates on body composition and strength and power-related performance in elite athletes Int J Sport Nutr Exerc Metab 2011 21 97 104 21558571 10.1123/ijsnem.21.2.97

[B68] Rodriguez NR Di Marco NM Langley S American Dietetic A, Dietitians of C, American College of Sports M American College of Sports Medicine position stand. Nutrition and athletic performance Med Sci Sports Exerc 2009 41 709 731 19225360 10.1249/MSS.0b013e31890eb86

[B69] Burke LM Loucks AB Broad N Energy and carbohydrate for training and recovery J Sports Sci 2006 24 675 685 10.1080/02640410500482602 16766497

[B70] Paddon-Jones D Westman E Mattes RD Wolfe RR Astrup A Westerterp-Plantenga M Protein, weight management, and satiety Am J Clin Nutr 2008 87 1558S 1561S 18469287 10.1093/ajcn/87.5.1558S

[B71] Dirlewanger M di Vetta V Guenat E Battilana P Seematter G Schneiter P Jequier E Tappy L Effects of short-term carbohydrate or fat overfeeding on energy expenditure and plasma leptin concentrations in healthy female subjects Int J Obes Relat Metab Disord 2000 24 1413 1418 10.1038/sj.ijo.0801395 11126336

[B72] Chin-Chance C Polonsky KS Schoeller DA Twenty-four-hour leptin levels respond to cumulative short-term energy imbalance and predict subsequent intake J Clin Endocrinol Metab 2000 85 2685 2691 10946866 10.1210/jcem.85.8.6755

[B73] Jenkins AB Markovic TP Fleury A Campbell LV Carbohydrate intake and short-term regulation of leptin in humans Diabetologia 1997 40 348 351 10.1007/s001250050686 9084976

[B74] Dulloo AG Jacquet J Girardier L Poststarvation hyperphagia and body fat overshooting in humans: a role for feedback signals from lean and fat tissues Am J Clin Nutr 1997 65 717 723 9062520 10.1093/ajcn/65.3.717

[B75] Dulloo AG Jacquet J Montani JP How dieting makes some fatter: from a perspective of human body composition autoregulation Proc Nutr Soc 2012 71 379 389 10.1017/S0029665112000225 22475574

[B76] Jackman MR Steig A Higgins JA Johnson GC Fleming-Elder BK Bessesen DH MacLean PS Weight regain after sustained weight reduction is accompanied by suppressed oxidation of dietary fat and adipocyte hyperplasia Am J Physiol Regul Integr Comp Physiol 2008 294 R1117 1129 10.1152/ajpregu.00808.2007 18287221

[B77] Saarni SE Rissanen A Sarna S Koskenvuo M Kaprio J Weight cycling of athletes and subsequent weight gain in middleage Int J Obes 2006 30 1639 1644 10.1038/sj.ijo.0803325 16568134

